# *Caenorhabditis elegans* NMAD-1 functions as a demethylase for actin

**DOI:** 10.1093/jmcb/mjad008

**Published:** 2023-02-10

**Authors:** Yu Shi, Hui Yang, Jianping Ding

**Affiliations:** State Key Laboratory of Molecular Biology, Shanghai Institute of Biochemistry and Cell Biology, Center for Excellence in Molecular Cell Science, University of Chinese Academy of Sciences, Chinese Academy of Sciences, Shanghai 200031, China; School of Life Science and Technology, ShanghaiTech University, Shanghai 201210, China; State Key Laboratory of Molecular Biology, Shanghai Institute of Biochemistry and Cell Biology, Center for Excellence in Molecular Cell Science, University of Chinese Academy of Sciences, Chinese Academy of Sciences, Shanghai 200031, China; State Key Laboratory of Molecular Biology, Shanghai Institute of Biochemistry and Cell Biology, Center for Excellence in Molecular Cell Science, University of Chinese Academy of Sciences, Chinese Academy of Sciences, Shanghai 200031, China; School of Life Science and Technology, ShanghaiTech University, Shanghai 201210, China; School of Life Science, Hangzhou Institute for Advanced Study, University of Chinese Academy of Sciences, Hangzhou 310024, China

**Dear Editor**,

DNA N^6^-methyladenine (6mA) modifi-cation, which was found in bacteria, archaea, viruses, and eukaryotes ranging from unicellular protists, fungi, and ciliates to multicellular plants and animals, has been proposed to be potentially associated with some important biological processes such as gene expression regulation and oncogenic pathways (Wion and Casadesus, 2006; Boulias and Greer, 2022). Yet, recent studies highlighted the arguments on the rarity, source, and origin of 6mA in invertebrates and vertebrates (Boulias and Greer, 2022). Removal of 6mA was suggested to be achieved by AlkB family dealkylating enzymes including ALKBH1 and ALKBH4 in mammals and NMAD-1 in *Caenorhabditis elegans* (Greer et al., 2015; Wu et al., 2016; Xiao et al., 2018; Xie et al., 2018). Silencing genes encoding these candidate enzymes induced global 6mA accumulation *in vivo*; however, *in vitro* catalytic activities and specificities of these enzymes remain to be characterized. AlkB family members are Fe(II)/α-ketoglutarate (α-KG)-dependent dioxygenases, which catalyze demethylation of various substrates including DNAs, RNAs, and proteins (Li et al., 2013; Fedeles et al., 2015; Kweon et al., 2019). *C. elegans* NMAD-1 was shown to exhibit *in vitro* catalytic activity on DNA 6mA and to be essential for DNA repair and replication during meiosis in the germline, but its substrate was unidentified (Wang et al., 2019). Remarkably, ALKBH4, the closest mammalian ortholog of NMAD-1, was shown to mediate demethylation of DNA 6mA and Lys84 mono-methylated actin (K84me1), suggesting alternative functional roles (Li et al., 2013; Kweon et al., 2019). Intriguingly, ALKBH4-dependent actin K84me1 demethylation facilitates the binding of non-muscle myosin II to unmethylated actin and thus regulates actomyosin function, indicating an important role of ALKBH4 in cytokinesis (Li et al., 2013). Whether the actin methylation exists in *C. elegans* or other eukaryotes is unclear. The substrate specificity and catalytic mechanism of NMAD-1 also remain to be determined.

Here, we investigated the substrate preference, catalytic mechanism, and biological functions of NMAD-1. It was previously reported that NMAD-1 demethylates 6mA in hemi or dual methylated double-stranded DNA (dsDNA) and single-site methylated single-stranded DNA (ssDNA) (Greer et al., 2015). We found that NMAD-1 failed to interact with various DNA oligos with different sequences and structures including ssDNAs, dsDNAs, and dsDNAs with a single mismatch at 6mA site in our electrophoretic mobility shift assay (Supplementary Table S1 and Figure S1A) and microscale thermophoresis (MST) measurement (Supplementary Figure S1B). Although no detectable binding affinity was observed, we cannot rule out the possibility that NMAD-1 may bind to other DNA substrates with different sequence(s) and/or structure(s) under different conditions.

We then sought for the possibility that NMAD-1 is associated with methylated actin in *C. elegans*. Mass spectrometric analysis of endogenous actin extracted from *C. elegans* cells identified a mono-methylation modification on Lys85 (equivalent to bovine and human Lys84) as a type of post-translational modification in *C. elegans* (Supplementary Figure S1C), which was further confirmed by western blot analysis using a previously reported antibody that specifically recognizes actin K84me1 (Figure 1A; Li et al., 2013). The K85me1 level was significantly increased in endogenous actin extracted from NMAD-1-deficent worm cells (*ok3133*), suggesting that NMAD-1 is associated with the demethylation of actin K85me1 in *C. elegans* (Figure 1A). Since nematode, human, and bovine actins share nearly identical sequences (Supplementary Figure S2), we then performed *in vitro* demethylation assay using recombinant NMAD-1 protein and commercial bovine actin extraction, and found that NMAD-1 directly catalyzed the demethylation of actin K84me1 in a dose-dependent mode (Figure 1B). Furthermore, recombinant NMAD-1 can physically bind to recombinant unmethylated bovine actin and the cofactor analog N-oxalylglycine (NOG) with dissociation constants (*K*_D_) of 10.7 ± 1.7 μM and 1.07 ± 0.11 μM, respectively (Figure 1C and D; Supplementary Table S2). Taken together, NMAD-1 directly interacts with actin and functions as a demethylase for actin K85me1 in *C. elegans*.

**Figure 1:**
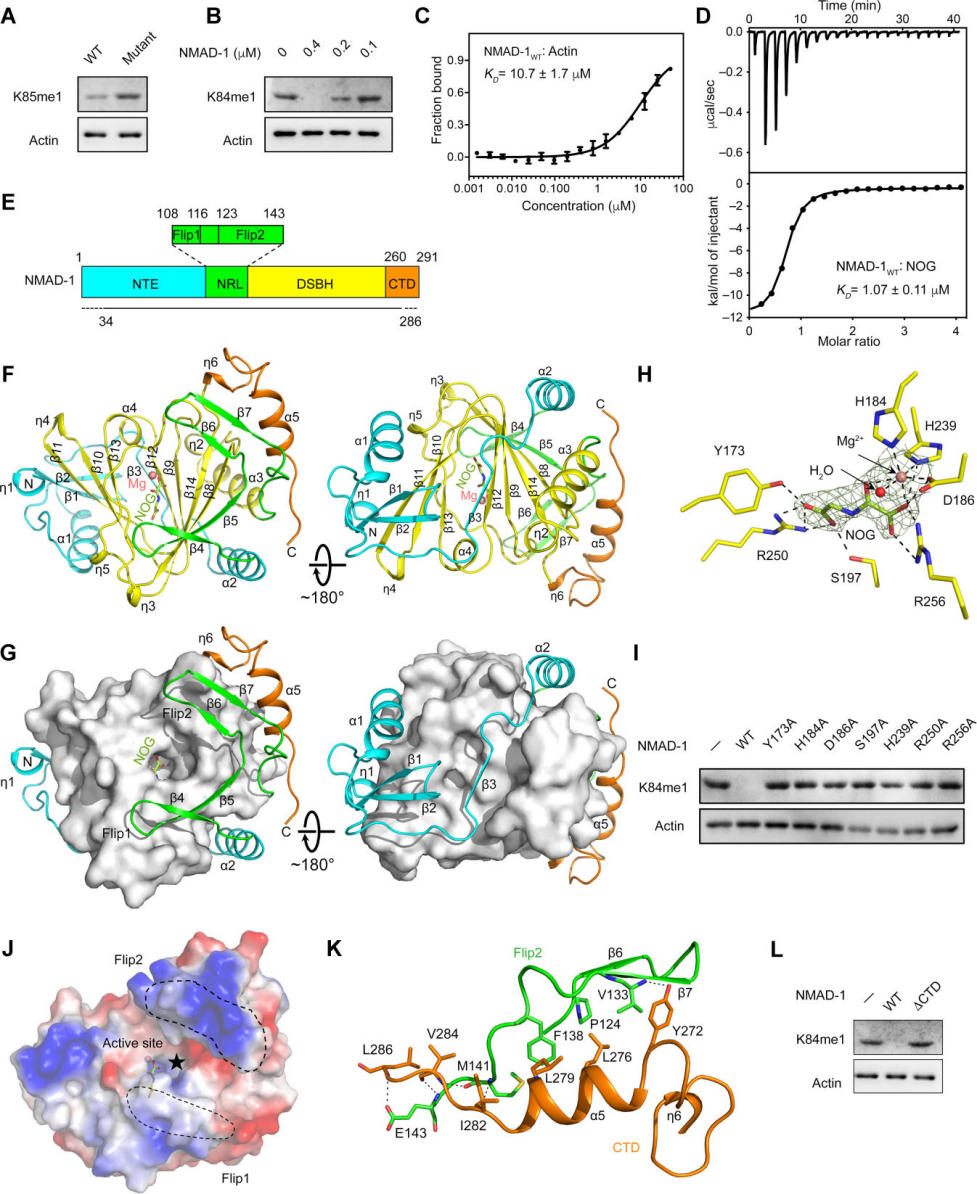
NMAD-1 mediates the demethylation of actin K85me1. (**A**) Methylation level of actin Lys85 in wild-type (WT) and NMAD-1-deficient (mutant) *C. elegans* cells. NMAD-1 depletion induced the accumulation of actin K85me1. (**B**) *In vitro* demethylation assay of NMAD-1 towards bovine actin. WT full-length NMAD-1 exhibited dose-dependent demethylation activity on actin K84me1. (**C**) MST measurement of the binding affinity between WT NMAD-1 and recombinant actin. (**D**) Isothermal titration calorimetry measurement of the binding affinity between WT NMAD-1 and NOG. (**E**) Domain organization of NMAD-1. The NTE, NRL, DSBH, and CTD are colored in cyan, green, yellow, and orange, respectively. The Flip1 and Flip2 motifs of the NRL domain are shown in a zoom-in box. Residues that were not modeled in the structure are indicated with dashed lines. (**F**) Ribbon representation of NMAD-1 in complex with Mg^2+^ and NOG. The color coding is the same as in panel **E**. NOG is shown in a stick model and Mg^2+^ is shown as a salmon ball. (**G**) The spatial connection of DSBH with NTE, NRL, and CTD. DSBH is shown as light gray surface, while the other elements are shown as colored ribbons. (**H**) Detailed interactions of key residues of NMAD-1 with NOG and Mg^2+^. For clarity, only the *2Fo*—*Fc* composite omit map for NOG and Mg^2+^ is shown (contoured at 1.5 σ level). (**I**) *In vitro* actin demethylation activity assay of mutations of key residues that are involved in the metal ion or NOG binding. (**J**) Electrostatic potential surface representation of NMAD-1 with the Flip1 and Flip2 motifs highlighted by dashed lines. (**K**) Detailed interactions between CTD and Flip2. Hydrogen-bonding interactions are indicated with dashed lines. (**L**) *In vitro* actin demethylation activity assay of WT and CTD-deleted (residues 1–259) NMAD-1. Deletion of the CTD abrogated the demethylation activity of NMAD-1. ΔCTD, deletion of the CTD.

To better understand the substrate recognition and catalytic mechanism of NMAD-1, we determined the crystal structure of NMAD-1 truncation (Δ1–31, hereafter referred to as NMAD-1_32–291_) in the presence of Mg^2+^ and NOG at 2.20 Å resolution (Figure 1E–G; Supplementary Table S3). The N-terminal flexible region (residues 1–31) was removed to facilitate the crystallization. The NMAD-1_32–291_ truncation retains comparable catalytic activity on actin K84me1 (Supplementary Figure S3A) and comparable binding affinity to both recombinant bovine actin (Supplementary Figure S3B) and NOG as the full-length NMAD-1 (Supplementary Figure S3C). NMAD-1_32–291_ was fused with a maltose binding protein (MBP) at the N-terminus to further facilitate the crystallization. In the crystal structure, MBP contributes to crystal packing but does not interfere with the cofactor binding or constrain the overall structure of NMAD-1 (Supplementary Figures S4 and S5).

NMAD-1 is comprised of an N-terminal extension (NTE) domain, a potential nucleotide-recognition lid (NRL) domain, a double-stranded β helix (DSBH) domain, and a C-terminal domain (CTD); the NRL domain contains the Flip1 and Flip2 motifs (Figure 1E–G). Similar to other Fe(II)/α-KG-dependent dioxygenases, the DSBH domain of NMAD-1 acts as the catalytic core, which consists of a seven-stranded jelly-roll fold (β8–β14), a large α-helix α3, a small α-helix α4, and four 3_10_ helices (η2–η5) (Figure 1F). The seven β-strands (β8–β14) fold into two β-sheets, which sandwich Mg^2+^ and NOG at the active center. The major β-sheet is formed by four antiparallel β-strands (β8–β14–β9–β12), and the minor β-sheet by three antiparallel β-strands (β13–β10–β11). The helices η3 and η5 and helices η4 and α4 locate on two sides of the jelly-roll fold, respectively. The large α-helix α3, together with the small 3_10_ helix η2, stacks and buttresses the major β-sheet from the back. The DSBH domain is surrounded by the NTE domain, the NRL domain, and the CTD, with the NRL domain and the CTD packing on one side and the NTE domain on the opposite side (Figure 1F and G).

Mg^2+^ and NOG bind to the active site with well-defined electron density (Figure 1H). Mg^2+^ is coordinated in an octahedral geometry by the sidechains of residues His184, Asp186, and His239 (so-called HxD…H motif) as well as two oxygen atoms of NOG and a water molecule (Figure 1H). Apart from the metal ion, the recognition and binding of NOG are achieved by hydrogen bonds contributed by the sidechains of Tyr173, Asp186, Ser197, and His239 and salt bridges by the sidechains of Arg250 and Arg256 (Figure 1H). Sequence alignment reveals that all of these key residues are highly conserved among NMAD-1 orthologs in different species (Supplementary Figure S6) as well as in other Fe(II)/α-KG-dependent dioxygenases (Supplementary Figure S7; Herr and Hausinger, 2018; Xu et al., 2021). We performed alanine substitution of these key residues involved in the metal ion and NOG binding and found that all single mutations dramatically reduced the demethylation activity on actin (Figure 1I), highlighting a conserved active site.

The NRL domain, especially the Flip1 and Flip2 motifs, adopts variable conformation and plays an essential role in the substrate binding among different AlkB family members (Xu et al., 2021). In NMAD-1, the NRL domain presents a unique architecture distinct from human AlkB members (Figure 1J; Supplementary Figures S8 and S9). The connecting strand β5 between Flip1 and Flip2 antiparallelly packs to β8 to extend the major β-sheet (Figure 1F). Flip1 consists of a short β-strand β4 (three residues) and the adjacent loops and is exposed to the solvent for substrate recognition. No basic residue was found in this region (Figure 1J; Supplementary Figures S6, S8, and S9), which may rationalize the substrate preference of NMAD-1 for actin over nucleic acid. Flip2 is mainly composed of the β6–β7 hairpin and protrudes from the surface to form a positive ‘horn’ (Figure 1G and J; Supplementary Figures S6, S8, and S9), which may also contribute to the substrate selectivity.

Notably, the CTD locates beyond and stabilizes Flip2 by hydrophobic contacts involving Tyr272, Leu276, and Leu279 of the CTD as well as Pro124, Val133, and Phe138 of Flip2 (Figure 1K). Most of these residues are highly conserved across NMAD-1 orthologs except for Tyr272 (Supplementary Figure S6), implying a potentially conserved interaction mode between Flip2 and the CTD. In addition, the mainchains of Ile282, Val284, and Leu286 of the CTD, together with the sidechain of Tyr272 of the CTD, form several hydrogen bonds with the mainchains of Val133, Met141, and Glu143 and the sidechain of Glu143 of Flip2 (Figure 1K). Deletion of the CTD dramatically reduced *in vitro* demethylation activity of NMAD-1 (Figure 1L) and disrupted the binding with actin (Supplementary Figure S10A). However, the CTD-deleted NMAD-1 retained the NOG binding affinity, suggesting that the DSBH domain maintains the structural integrity in the absence of the CTD (Supplementary Figure S10B). These results indicate that the CTD is essential for the catalytic activity through the formation of substrate recognition interface with Flip2. Further structural analysis also revealed the shape and charge complementarity between the K84-containing surface of actin and the surface of NMAD-1 (Supplementary Figure S11), implying that NMAD-1 has higher specificity and selectivity for actin K84me1 substrate than other proteins.

In summary, we found that NMAD-1 is able to directly bind to and demethylate *C. elegans* actin K85me1 (equivalent to bovine and human actin K84me1) *in vitro* and its deficiency dramatically increases actin methylation level *in vivo*. Our structural and biochemical data revealed that NMAD-1 assumes a typical DSBH domain and contains a highly conserved catalytic site for the metal ion and cofactor binding and actin demethylation. The unique NRL domain and the CTD play critical roles in the substrate selectivity. Our research highlights NMAD-1 as an actin K85me1 demethylase in nematode and provides molecular insights into the catalytic mechanism, which paves the way for further investigation of the functional roles and mechanisms of NMAD-1 in actin K85me1 maintenance and DNA replication and repair. Whether the binding of NMAD-1 to DNA repair and replication machinery relies on actin K85me1 requires further investigation.

*[Supplementary material is available at Journal of Molecular Cell Biology online. The crystal structure of NMAD-1 has been deposited in the Protein Data Bank with accession number 8H68. We thank the staff of the BL19U1 beamline of the National Center for Protein Science in Shanghai (NCPSS) for technical assistance in diffraction data collection and other members of our group for helpful discussion. We are grateful to Dr Yidong Shen (Center for Excellence in Molecular Cell Science, Chinese Academy of Sciences) for providing the C. elegans strains. This work was supported by grants from the National Key Research and Development Program of China (2020YFA0803200 and 2020YFA0509000). Y.S. carried out the protein expression and purification, crystallization, and functional studies. H.Y. carried out the structural determination. J.D. and H.Y. conceived the study, designed the experiments, interpreted the data, and wrote the manuscript.]*

## Supplementary Material

mjad008_Supplemental_File
