# Supplementary material for: Caenorhabditis elegans NMAD-1 functions as a demethylase for actin
Source: J Mol Cell Biol. 2023 Feb 10;15(1):mjad008. doi: 10.1093/jmcb/mjad008 (PMC10278977; doi:10.1093/jmcb/mjad008)
Supplement: mjad008_Supplemental_File [file mjad008_supplemental_file.pdf]

Supplementary Materials and Methods

Protein expression and purification

The full-length *nmad-1* gene was cloned into pET-28a vector with a His_6-sumo tag attached at the N-terminal. The plasmid was transformed into *E. coli* BL21 (DE3) CodonPlus strain. The transformed bacterial cells were grown in 2xYT medium at 37 °C until OD_{600} reached 1.2-1.4 and then induced with 0.5 mM isopropyl β-D-1-thiogalactopyranoside (IPTG) at 16 °C for 18 hours. The cells were collected and resuspended in buffer A (20 mM Tris-HCl, pH 8.0, and 500 mM NaCl) supplemented with 2 mM β-mercaptoethanol (β-ME) and 1 mM phenylmethylsulfonyl fluoride (PMSF) and then lysed on ice by high-pressure cell disrupter. The cell debris was separated by centrifugation and discarded. The supernatant containing recombinant NMAD-1 protein was collected and purified by affinity chromatography using Ni-NTA beads (Qiagen) pre-equilibrated with buffer A and eluted by buffer A supplemented with 300 mM imidazole. The elution was dialyzed against buffer A, incubated with homemade ULP1 protease overnight at 4 °C, and then reloaded onto a Ni-NTA column to remove the His_6-sumo tag. The NMAD-1 protein was further purified by gel filtration using Superdex G200 10/300 column (Cytiva) pre-equilibrated with buffer B (20 mM HEPES, pH 8.0, 200 mM NaCl, and 2 mM DTT). The purified NMAD-1 protein was of high purity (above 95%) as analyzed by SDS-PAGE.

To facilitate crystallization, maltose binding protein (MBP) followed by an AAAA linker was fused to the N-terminal of an NMAD-1 truncation (residues 32-291). The genes of the NMAD-1 truncation (residues 32-291) with or without MBP were inserted into the same vector, respectively. NMAD-1 mutations were generated using the QuikChange Site-Directed Mutagenesis kit (Stratagene) and verified by sequencing. The NMAD-1 truncations and mutations, and the MBP-fused NMAD-1 truncation, were expressed and purified using the same methods as described above.

Recombinant bovine β-actin was prepared using a common unfolding and refolding method. The *actin* gene was inserted into pET-28a vector and attached with a 6xHis tag at the C-terminal. The plasmid was transformed into *E. coli* BL21 (DE3) CodonPlus strain. The transformed bacterial cells were grown in LB medium at 37 °C to an OD_{600} of 0.6-0.8, and then induced with 0.5 mM IPTG at 37 °C for 6 hours. The cells were collected, resuspended in buffer C (50 mM Tris-HCl, pH 7.5, 100 mM NaCl, and 1 mM EDTA) supplemented with 2 mM β-ME and 1 mM benzamidine, and then lysed on ice by high-pressure cell disrupter. The cell debris containing actin inclusion body was separated and collected by centrifugation. The pellet was resuspended and washed by buffer D (buffer C supplemented with 1% Triton X100) twice and further washed by buffer C one time. The inclusion body was then dissolved in buffer E (7 M guanidine hydrochloride, 20 mM Tris-HCl, pH 7.5, and 10 mM DTT) at room temperature for 1 hour. The clear supernatant containing denatured actin was collected by centrifugation, loaded on a Ni-excel column (Cytiva) pre-equilibrated with buffer F (8 M urea and 20 mM Tris-HCl, pH 7.5), washed with buffer F

supplemented with 30 mM imidazole and eluted by buffer F supplemented with 300 mM imidazole. The elution was successively dialyzed against buffer F with decreased concentrations of urea (6 M to 2 M) at 4 °C for 12 hours and then dialyzed against refolding buffer (20 mM Tris-HCl, pH 7.5, 2 mM GSH, 0.2 mM GSSG, 5 mM EDTA, 10 mM ATP, 50 mM glycine, 1% saccharose, and 0.1% PEG 6000) at 4 °C for 12 hours twice. The refolded actin was dialyzed in HBS buffer (20 mM HEPES, pH 7.4, and 100 mM NaCl) at 4 °C for 12 hours for microscale thermophoresis (MST) assay.

In vitro demethylation activity assay

The demethylase activity of NMAD-1 on actin-K84me1 was measured using commercial actin mixture extracted from bovines (Sangon Biotec). Briefly, 4 μg (2 μM) bovine actin mixture was incubated with NMAD-1 protein with varied concentrations (0.4 μM, 0.2 μM and 0.1 μM) in the reaction buffer (50 mM Tris-HCl, pH 7.4, 50 μM $(NH_4)_2Fe(SO_4)_2$, 1 mM α-KG, and 2 mM ascorbate). The demethylation reaction was performed at 37 °C for 1 hour and then stopped by adding 5xSDS loading buffer (10% SDS, 500 mM DTT, 50% glycerol, 500 mM Tris-HCl, and 0.05% bromophenol blue dye). The product was analyzed by Western blot with actin K84me1 antibody (Li et al., 2013). The NMAD-1 truncations and mutations, and the MBP-fused NMAD-1 truncation were measured by the same method. All experiments were performed at least three times.

Electrophoretic mobility shift assay (EMSA)

The purified NMAD-1 protein and the FAM-labeled DNA (0.5 μM) were mixed with varied molar ratios (1:1, 5:1, 10:1, and 20:1) and then incubated at 4 °C for 1 hour in the binding buffer (20 mM Tris-HCl, pH 7.4, 100 mM NaCl, 200 μM α-KG, 5 mM $MgCl_2$, and 10% glycerol). 20 μl mixture was run on 6% native PAGE at 4 °C and visualized by detection of FAM fluorescence using FUJIFILM FLA9000 (FUJIFILM). To generate dsDNA, 5'-FAM labeled DNA oligo was mixed with unmodified reverse complement oligo at 1:1 molar ratio, denatured at 95 °C for 10 min, and then cooled gradually to room temperature.

Previous studies found that 6mA was significantly enriched in AGAA and GAGG motifs in *C. elegans*, AGAAGAGGA motif in mouse, and [G/C] AGG[C/T] motif in human (Greer et al., 2015; Xiao et al., 2018; Kweon et al., 2019). Two 42 bp hemi-methylated dsDNA oligos were designed following the reported sequences to contain a 5'-(6mA)GAAG(6mA)GGA-3' motif (DNA1_ds) (Kweon et al., 2019) or a single 6mA site (DNA2_ds) (Greer et al., 2015), respectively. Two shorter 17 bp hemi-methylated dsDNA oligos were designed with a 5'-AG(6mA)A-3' motif (DNA3_ds) and a 5'-G(6mA)GG-3' motif (DNA4_ds), respectively. For mismatch oligos, a single mismatch was introduced at 6mA site which might facilitate the flip-out of 6mA and thus enhance the recognition ability. All DNA oligos used in the EMSA assay were listed in Table S1.

Isothermal titration calorimetry (ITC) measurement

For ITC measurement, the NMAD-1 protein and the cofactor analog N-oxalylglycine (NOG) were prepared in buffer containing 20 mM HEPES, pH 7.4, and 150 mM NaCl. The titration was performed using MicroCal PEAQ-ITC (Malvern Panalytical) at 25 °C. 250 μl of 50 μM NMAD-1 protein was added to the sample cell, while 500 μM NOG was filled into syringe. Each titration consisted of 20 injections, in which the first was set as 0.4 μl and the rest was set as 2 μl. The first injection was discarded to eliminate the effect of titrant diffusion across the syringe tip during the equilibration process. The dissociation constant (K_d), enthalpy (ΔH), entropy (ΔS), free energy (ΔG), and stoichiometry (n) were determined by fitting the integrated titration data using 'One Set of Sites' fitting model by a nonlinear least-squares method implemented in MicroCal PEAQ-ITC analysis software v1.1.0.1262 (Malvern). The NOG-binding affinities of the truncated or MBP-fused NMAD-1 protein were measured by the same method. MBP protein was used as a negative control and measured by the same method. The thermodynamic parameters were summarized in Table S2.

C. elegans strains and cultivation in liquid medium

The N2 Bristol wild-type and VC2552 [F07F7.7 (*ok3133*) III] (*nmad-1* mutant) strains were obtained from *Caenorhabditis* Genetics Center (CGC, University of Minnesota, Twin Cities, MN). For the wild-type strain, 5-8 large starved plates of *C. elegans* containing adult and young larvae were washed with S medium (100 mM NaCl, 4.4 mM K_2HPO_4, 44 mM KH_2PO_4, 5 mg/ml cholesterol, 500 mM EDTA, 250 mM $FeSO_4$, 100 mM $MnCl_2$, 100 mM ZnSO4, 15 mM $CuSO_4$, 30 mM $MgSO_4$, and 30 mM $CaCl_2$) and then transferred into 1 L S medium supplemented with *E. coli* OP50 to final concentration of 25 mg/ml. The cells were grown at 20 °C at the speed of 130 rpm for 8 hours followed by 240 rpm for 90 hours. The cells were harvested by centrifugation at 3000 g for 5 min and the worm pellets were frozen and stored at -80 °C. The *nmad-1* (*ok3133*) mutant cells were prepared using the same method as described above. The endogenous actin was extracted from the wild-type and mutant *C. elegans* cells as described previously (Ono and Pruyne, 2012).

Microscale thermophoresis (MST) assay

To determine the binding affinity to actin, the recombinant NMAD-1 and bovine actin proteins were prepared and measured in an MST buffer containing 20 mM HEPES, pH 7.4, 100 mM NaCl, and 0.05% P20 (Cytiva). The NMAD-1 protein was fluorescently labeled using Monolith NT Protein Labeling Kit RED-NHS (NanoTemper Technologies). The bovine actin protein was prepared in 16-step two-fold serial dilution with the final concentrations ranging from 50 μM to 0.001526 μM in the MST buffer. 10 μl actin

with varied concentrations was mixed with 10 μl of 500 nM fluorescently labeled NMAD-1 and then incubated on ice for 30 min. To determine the binding affinity to DNA substrate, dsDNA DNA1_ds was two-fold serial dilution with the final concentrations ranging from 100 μM to 0.003125 μM in the MST buffer supplemented with 200 μM α-KG and 5 mM $MgCl_2$. 10 μl of 500 nM fluorescently labeled NMAD-1 in the same modified MST buffer was mixed with 10 μl DNA1_ds with different concentrations and then incubated on ice for 1 hour.

The samples were subsequently loaded into NanoTemper standard treated capillaries and the microscale thermophoresis was measured using 40% IR-Laser Power on a Monolith NT.115 (NanoTemper Technologies) at 25 °C. The data were analyzed by Monolith Affinity Analysis 3 software (NanoTemper Technologies). The dissociation constant (K_d) was calculated by plotting the fluorescence data against the concentration of actin (Nanotemper Monolith Technical Manual).

Crystallization, diffraction data collection, and structure determination

NMAD-1 is a member of the Fe(II)/α-KG-dependent dioxygenase superfamily. We replaced the Fe(II) and α-KG with Mg^{2+} and NOG to keep NMAD-1 in a catalytically inactive state. Prior to crystallization, the MBP-fused NMAD-1 truncation (residues 32-291) was mixed with 5 mM $MgCl_2$ and 5 mM NOG at a concentration of 16 mg/ml. Crystallization was carried out using the hanging drop vapor diffusion method at 16 °C by mixing equal volumes of protein solution and reservoir solution. Crystals were grown in the solution containing 0.1 M HEPES, pH 7.5, 10% (w/v) PEG 6000, and 10% (v/v) MPD. The crystals were cryoprotected by reservoir solution supplemented with 20% glycerol, and then flash-cooled into liquid nitrogen. Diffraction data were collected at 100 K at BL19U1 of National Center for Protein Science in Shanghai (NCPSS), and processed with XDS (Kabsch, 2010). Statistics of the diffraction data are summarized in Table S3. The crystal structure of NMAD-1 bound with Mg^{2+} and NOG was solved by the molecular replacement method using the structure model predicted by AlphaFold2 as the search model (Tunyasuvunakool et al., 2021). Model building was performed with Coot (Emsley and Cowtan, 2004) and structure refinement was carried out using Phenix (Adams et al., 2010). The asymmetric unit contains one MBP-NMAD-$1_{32\text{-}291}$ molecule. All residues of NMAD-$1_{32\text{-}291}$ were defined with unambiguous electron density except for residue 32 at the N-terminus and residues 287-291 at the C-terminus. Structural analysis was carried out using programs in CCP4 suite (Winn et al., 2011). Molecular graphics figures were generated using Pymol (www.pymol.org). Statistics of the structure refinement and the quality of final structure model are also summarized in Table S3.

Supplementary References

Adams, P.D., Afonine, P.V., Bunkoczi, G., et al. (2010). PHENIX: a comprehensive Python-based system for macromolecular structure solution. Acta Crystallogr D Biol Crystallogr *66*, 213-221.

Emsley, P., and Cowtan, K. (2004). Coot: model-building tools for molecular graphics. Acta Crystallogr D Biol Crystallogr *60*, 2126-2132.

Greer, E.L., Blanco, M.A., Gu, L., et al. (2015). DNA methylation on N6-adenine in *C. elegans*. Cell *161*, 868-878.

Kabsch, W. (2010). Xds. Acta Crystallogr D Biol Crystallogr *66*, 125-132.

Kaur, S., Tam, N.Y., McDonough, M.A., et al. (2022). Mechanisms of substrate recognition and N6-methyladenosine demethylation revealed by crystal structures of ALKBH5-RNA complexes. Nucleic Acids Res *50*, 4148-4160.

Kweon, S.-M., Chen, Y., Moon, E., et al. (2019). An adversarial DNA N6-methyladenine-sensor network preserves polycomb silencing. Mol Cell *74*, 1138-1147.e1136.

Li, M.M., Nilsen, A., Shi, Y., et al. (2013). ALKBH4-dependent demethylation of actin regulates actomyosin dynamics. Nat Commun *4*, 1832.

Ma, L., Lu, H., Tian, Z., et al. (2022). Structural insights into the interactions and epigenetic functions of human nucleic acid repair protein ALKBH6. J Biol Chem *298*, 101671.

Ono, S., and Pruyne, D. (2012). Biochemical and cell biological analysis of actin in the nematode *Caenorhabditis elegans*. Methods *56*, 11-17.

Pastore, C., Topalidou, I., Forouhar, F., et al. (2012). Crystal structure and RNA binding properties of the RNA recognition motif (RRM) and AlkB domains in human AlkB homolog 8 (ABH8), an enzyme catalyzing tRNA hypermodification. J Biol Chem *287*, 2130-2143.

Rebowski, G., Boczkowska, M., Drazic, A., et al. (2020). Mechanism of actin N-terminal acetylation. Sci Adv *6*, eaay8793.

Sundheim, O., Vagbo, C.B., Bjoras, M., et al. (2006). Human ABH3 structure and key residues for oxidative demethylation to reverse DNA/RNA damage. EMBO J *25*, 3389-3397.

Tunyasuvunakool, K., Adler, J., Wu, Z., et al. (2021). Highly accurate protein structure prediction for the human proteome. Nature *596*, 590-596.

Wang, G., He, Q., Feng, C., et al. (2014). The atomic resolution structure of human AlkB homolog 7 (ALKBH7), a key protein for programmed necrosis and fat metabolism. J Biol Chem *289*, 27924-27936.

Winn, M.D., Ballard, C.C., Cowtan, K.D., et al. (2011). Overview of the CCP4 suite and current developments. Acta Crystallogr D Biol Crystallogr *67*, 235-242.

Xiao, C.L., Zhu, S., He, M., et al. (2018). N(6)-methyladenine DNA modification in the human genome. Mol Cell *71*, 306-318 e307.

Yi, C., Chen, B., Qi, B., et al. (2012). Duplex interrogation by a direct DNA repair protein in search of base damage. Nat Struct Mol Biol *19*, 671-676.

Zhang, M., Yang, S., Nelakanti, R., et al. (2020). Mammalian ALKBH1 serves as an N(6)-mA demethylase of unpairing DNA. Cell Res *30*, 197-210.

Zhang, X., Wei, L.H., Wang, Y., et al. (2019). Structural insights into FTO's catalytic mechanism for the demethylation of multiple RNA substrates. Proc Natl Acad Sci U S A *116*, 2919-2924.

Supplementary Table S1. DNA oligos used for EMSA assay

Oligos	Sequence	length	Structure
DNA1_ds	F: 5'-FAM-GATGCAAGCATCAGCAAC(6mA)GAAG(6mA)GGATCTCAGGTGCAGCGC-3'<br>R: 5'-GCGCTGCACCTGAGATCCTCTTCTGTTGCTGATGCTTGCATC-3'	42 bp	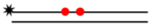
DNA1_mismatch	F: 5'-FAM-GATGCAAGCATCAGCAAC(6mA)GAAG(6mA)GGATCTCAGGTGCAGCGC-3'<br>R: 5'-GCGCTGCACCTGAGATCCTCTTCAGTTGCTGATGCTTGCATC-3'	42 bp	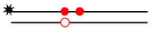
DNA1_ss	F: 5'-FAM-GATGCAAGCATCAGCAAC(6mA)GAAG(6mA)GGATCTCAGGTGCAGCGC-3'	42 nt	
DNA2_ds	F: 5′-FAM-GGGAATTTCCCGGCGATTTG(6mA)TCAAATCGCCGGGAAATTCCC-3′<br>R: 5'-GGGAATTTCCCGGCGATTTGATCAAATCGCCGGGAAATTCCC-3'	42 bp	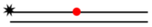
DNA2_mismatch	F: 5′-FAM-GGGAATTTCCCGGCGATTTG(6mA)TCAAATCGCCGGGAAATTCCC-3′<br>R: 5'-GGGAATTTCCCGGCGATTTGAACAAATCGCCGGGAAATTCCC-3'	42 bp	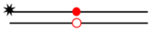
DNA2_ss	F: 5′-FAM-GGGAATTTCCCGGCGATTTG(6mA)TCAAATCGCCGGGAAATTCCC-3′	42 nt	
DNA3_ds	F: 5-FAM-GCAATAAG(6mA)AGTTTATC-3'<br>R: 5'-GATAAACTTCTTATTGC-3'	17 bp	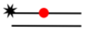
DNA3_mismatch	F: 5'-FAM-GCAATAAG(6mA)AGTTTATC-3'<br>R: 5'-GATAAACTACTTATTGC-3'	17 bp	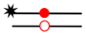
DNA3_ss	F: 5'-FAM-GCAATAAG(6mA)AGTTTATC-3'	17 nt	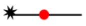
DNA4_ds	F: 5'-FAM-TCAAATCG(6mA)GGTTCCCA-3'<br>R: 5'-TGGGAACCTCGATTTGA-3'	17 bp	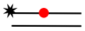
DNA4_mismatch	F: 5'-FAM-TCAAATCG(6mA)GGTTCCCA-3'<br>R: 5'-TGGGAACCACGATTTGA-3'	17 bp	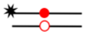
DNA4_ss	F: 5'-FAM-TCAAATCG(6mA)GGTTCCCA-3'	17 nt	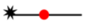

Supplementary Table S2. Thermodynamic parameters obtained from ITC assay

Protein	Ligand	K_d (μM)	N	ΔH (kcal/mol)	TΔS (kcal/mol)	ΔG (kcal/mol)	Figure
NMAD-1_{WT}	NOG	1.07 ± 0.11	0.66 ± 0.01	−11.60 ± 0.22	−3.44	−8.15	1D
NMAD-$1_{32\text{-}291}$	NOG	1.25 ± 0.10	0.84 ± 0.01	−10.20 ± 0.15	−2.11	−8.05	S3C
MBP-NMAD-$1_{32\text{-}291}$	NOG	1.36 ± 0.13	0.85 ± 0.01	−10.00 ± 0.18	−2.03	−8.01	S5C
NMAD-$1_{\Delta CTD}$	NOG	1.68 ± 0.01	0.72 ± 0.01	−7.29 ± 0.15	0.59	−7.88	S10B
MBP	NOG	ND	ND	ND	ND	ND	S5D
NMAD-$1_{32\text{-}291}$	MBP	ND	ND	ND	ND	ND	S4B

Supplementary Table S3. Summary of diffraction data and structure refinement statistics

NMAD-1-Mg^{2+}-NOG			
**Data collection**		**Refinement**	
Wavelength (Å)	0.9791	Resolution (Å)	50.0-2.20
Space group	$P2_12_12_1$	Reflections	
Cell parameters		Working set	29,443
a, *b*, *c* (Å)	50.03, 91.87, 133.30	Test set	1,987
α, β, γ (°)	90, 90, 90	R_{work}/R_{free} (%)[c]	18.3/24.2
Resolution (Å)	75.6-2.20 (2.28-2.20) [a]	No. of atoms	
Observed reflections	100,136 (9,976)	Protein	4,916
Unique reflections	31,436 (3,113)	Ligand	86
Average $I/\sigma(I)$	11.2 (2.2)	Water	197
Average redundancy	3.2 (3.2)	Wilson B-factor (Å^2)	37.1
Completeness (%)	98.2 (98.6)	Average B-factor (Å^2)	
R_{merge} (%)[b]	7.8 (53.4)	Protein	40.0
$CC_{1/2}$	0.995 (0.814)	Ligand	38.2
		Water	39.2
		R.m.s. deviations	
		Bond lengths (Å)	0.009
		Bond angles (°)	0.93
		Ramachandran plot (%)	
		Favored	98.1
		Allowed	1.9
		Outliers	0.0

[a]Numbers in parentheses represent the highest resolution shell.

[b]Rmerge = ∑hkl∑i|Ii(hkl)−<I(hkl)>|/∑hkl∑iIi(hkl).

[c]R = ∑hkl||Fo|−|Fc||/∑hkl|Fo|.

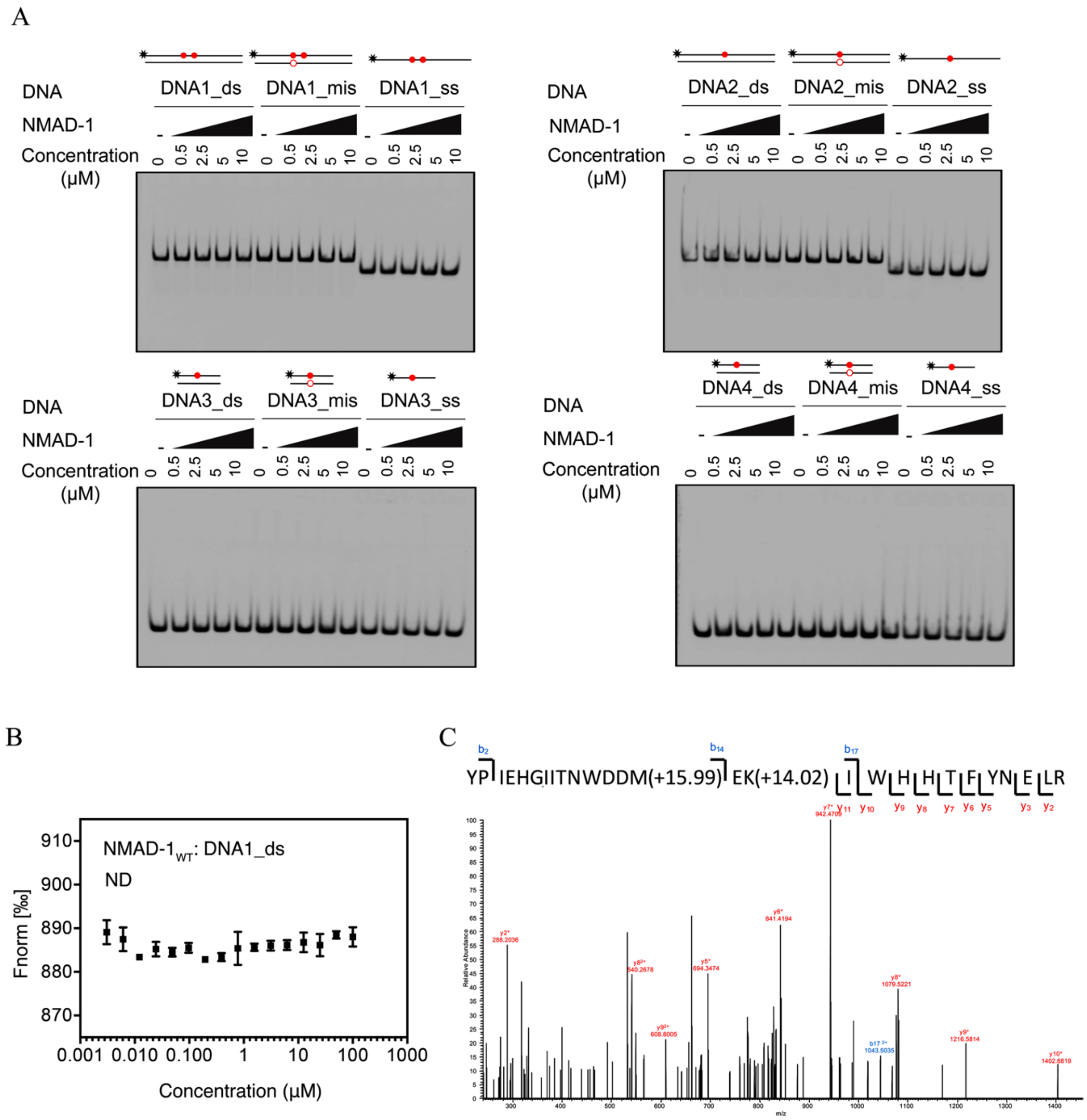

Supplementary Figure S1. NMAD-1 shows no detectable interactions with different DNA oligos but is associated with actin demethylation. (A) Electrophoretic mobility shift assay (EMSA) was performed to detect the interactions between NMAD-1 and different DNA oligos. 0.5 μM 5' FAM labeled DNA oligos were incubated with increasing concentrations of NMAD-1 protein as indicated. (**B)** The binding affinity between NMAD-1 and dsDNA substrate DNA1_ds was measured by MST. ND, no detected. **(C)** Mass spectrometric analysis reveals that actin Lys85 (equivalent to Lys84 in bovine and human) is mono-methylated in *C. elegans*.

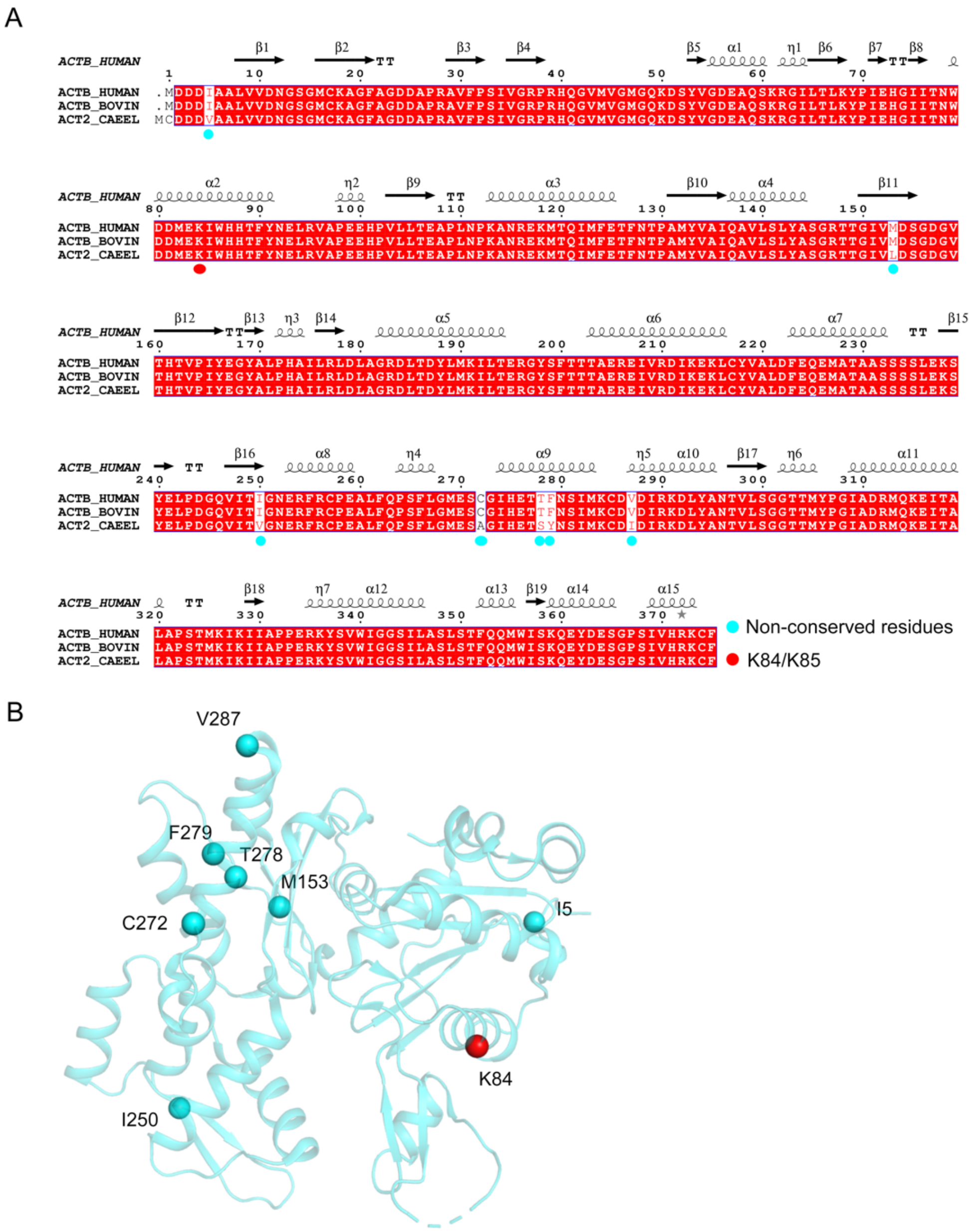

Supplementary Figure S2. Actin is highly conserved in human, bovine, and nematode. (A) Structure-based sequence alignment of β-actin orthologs in human, bovine, and nematode. Secondary structural elements of human β-actin are shown above the alignment. Actin is highly conserved in these species, and the mono-methylation site (Lys84 in human and bovine and Lys85 in nematode) is strictly conserved. **(B)** Ribbon diagram of the overall structure of human β-actin (PDB code 6NBW) (Rebowski et al., 2020). The variant residues Ile5, Met153, Ile250, Cys272, Thr278, Phe279, and Val287 are shown in cyan spheres and the invariant Lys84 is shown in red sphere. The variant residues are located far away from Lys84, implying that these residues are unlikely to have direct impacts on the methylation and demethylation of Lys84 in human and bovine and Lys85 in nematode.

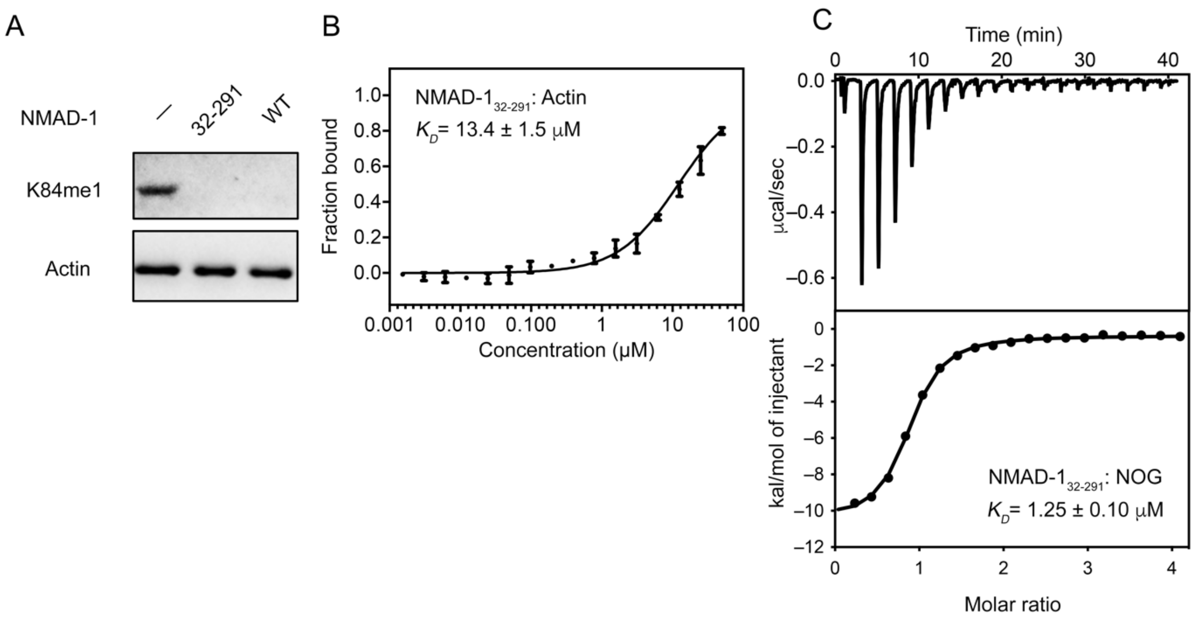

Supplementary Figure S3. **The NMAD-1$_{32\text{-}291}$ truncation retains comparable enzymatic activity. (A)** *In vitro* demethylation assay of the wild-type and truncated NMAD-1 on comemerical actin extraction from bovines. **(B)** The binding affinity between NMAD-1$_{32\text{-}291}$ and the recombinant actin measured by microscale thermophoresis (MST). **(C)** The binding affinity between NMAD-1$_{32\text{-}291}$ and NOG measured by isothermal titration calorimetry (ITC).

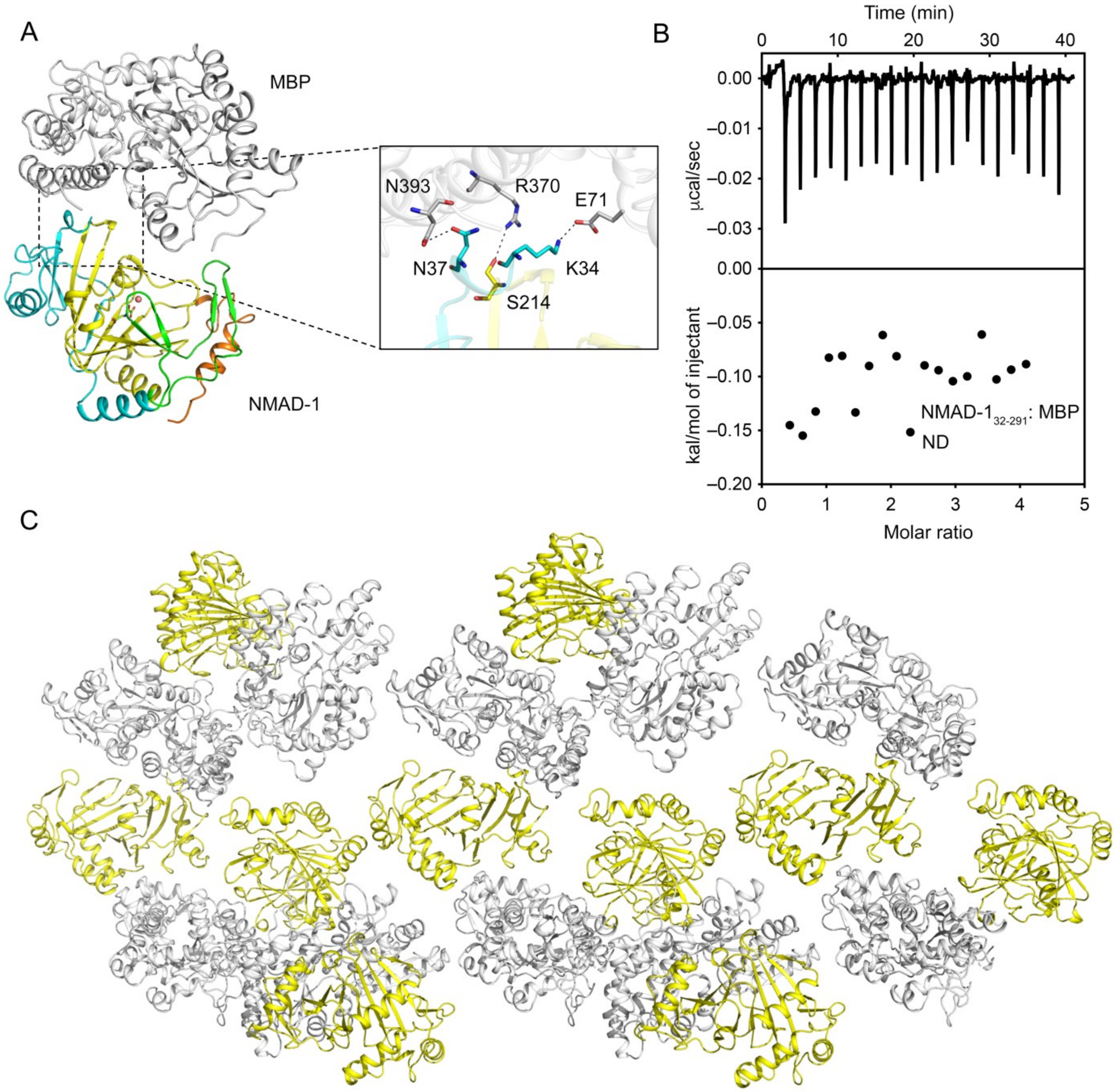

Supplementary Figure S4. **MBP has no constraint on the overall structure of NMAD-1. (A)** The fused MBP is located at one side of NMAD-$1_{32\text{-}291}$ and has few hydrophilic contacts with Lys34, Asn37 and Ser214 of NMAD-$1_{32\text{-}291}$ (zoom-in panel), resulting in a relatively narrow surface on NMAD-1 for substrate binding. **(B)** MBP has no detectable binding with NMAD-$1_{32\text{-}291}$ as measured by ITC. **(C)** Crystal packing of MBP-NMAD-$1_{32\text{-}291}$ shown in ribbon representation. The MBP is colored in light grey and NMAD-$1_{32\text{-}291}$ is colored in yellow. Crystal packing is mainly contributed by MBP-MBP interactions but not NMAD-$1_{32\text{-}291}$. Thus, MBP has no constraint on the overall structure of NMAD-1.

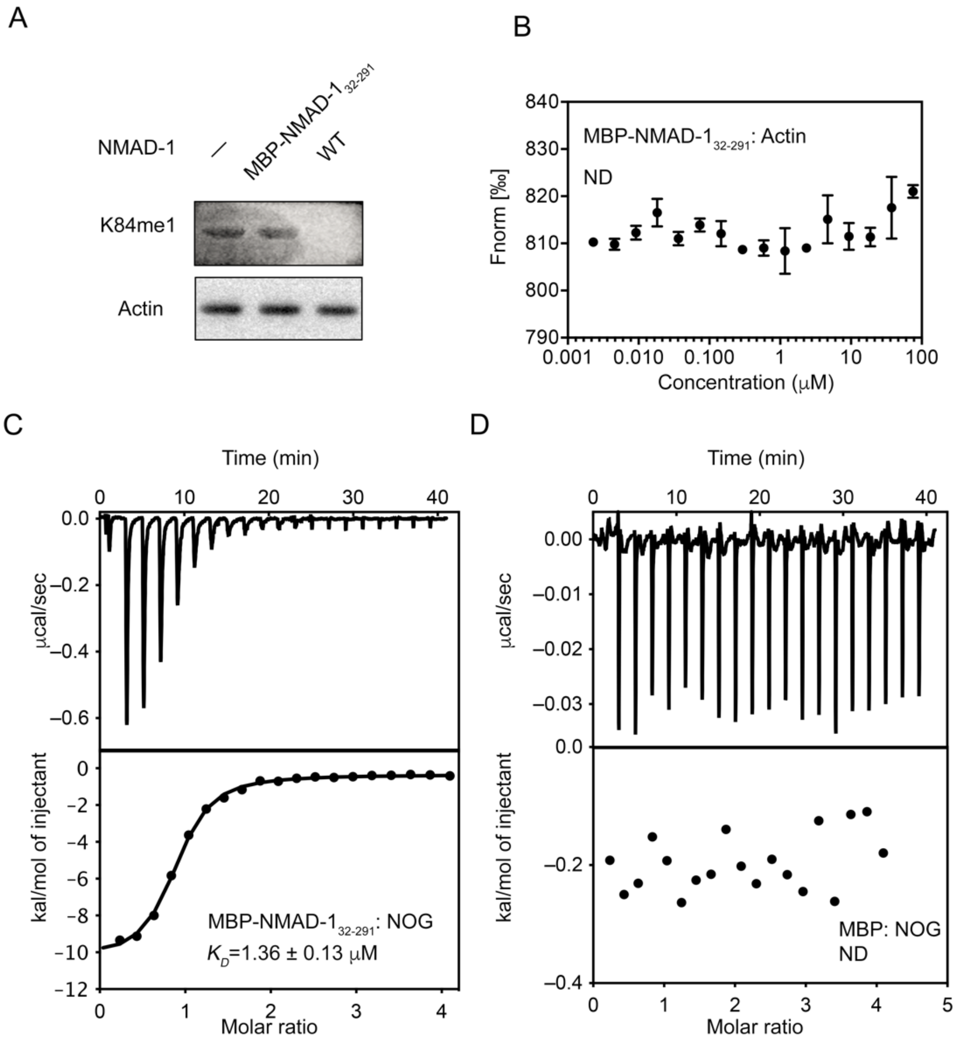

Supplementary Figure S5. **The fused MBP appears to prevent the substrate binding, but does not interfere the cofactor binding. (A)** The MBP fused NMAD-$1_{32\text{-}291}$ exhibited no *in vitro* catalytic activity on bovine actin mixture, which is consistent with the structural data showing that the fused MBP sterically blocks the actin binding (Supplementary Figure S4A). **(B)** Fusion of MBP with NMAD-$1_{32\text{-}291}$ impaired its binding to unmethylated recombinant bovine actin as measured by MST. **(C)** ITC measurement of the binding affinity between MBP-NMAD-$1_{32\text{-}291}$ and NOG. MBP-NMAD-$1_{32\text{-}291}$ exhibited relatively higher NOG binding affinity (K_d of 1.36 ± 0.13 μM, this panel) than wild-type full-length NMAD-1 (WT) (K_d of 1.07 ± 0.11 μM) (Figure 1D). **(D)** MBP showed no detectable binding with NOG as measured by ITC. These results suggest that the MBP fusion has no effect on the binding of NMAD-$1_{32\text{-}291}$ with NOG.

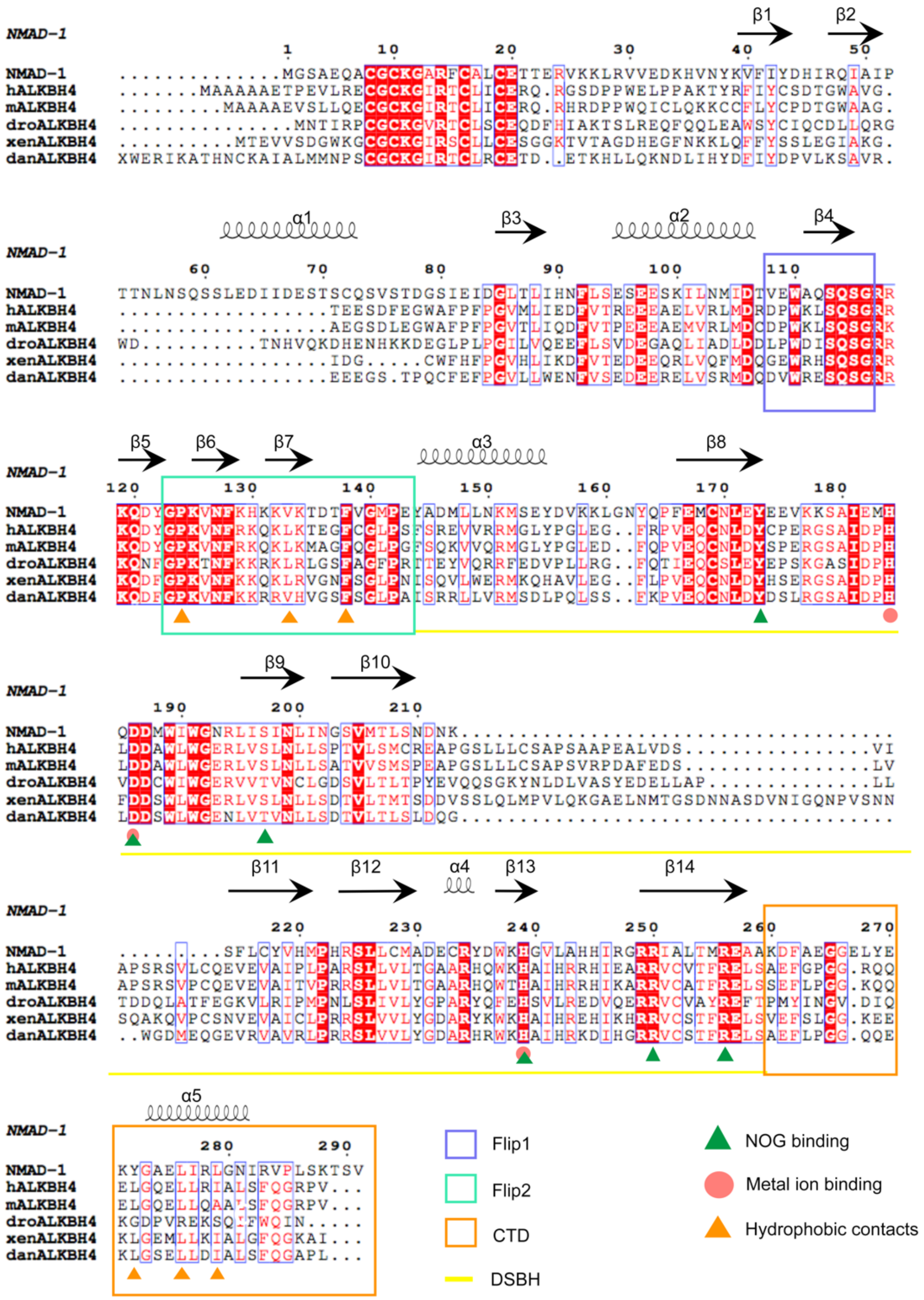

Supplementary Figure S6. **Structure-based sequence alignment of NMAD-1 orthologs from different species.** Secondary structural elements are represented according to the structure of NMAD-$1_{32\text{-}291}$. The key residues participating in the metal ion binding and NOG binding are indicated by salmon balls and green triangles, respectively. The Flip1 and Flip2 motifs and the CTD domain are highlighted with slate, green cyan, and orange boxes, respectively. The DSBH domain is underlined in yellow.

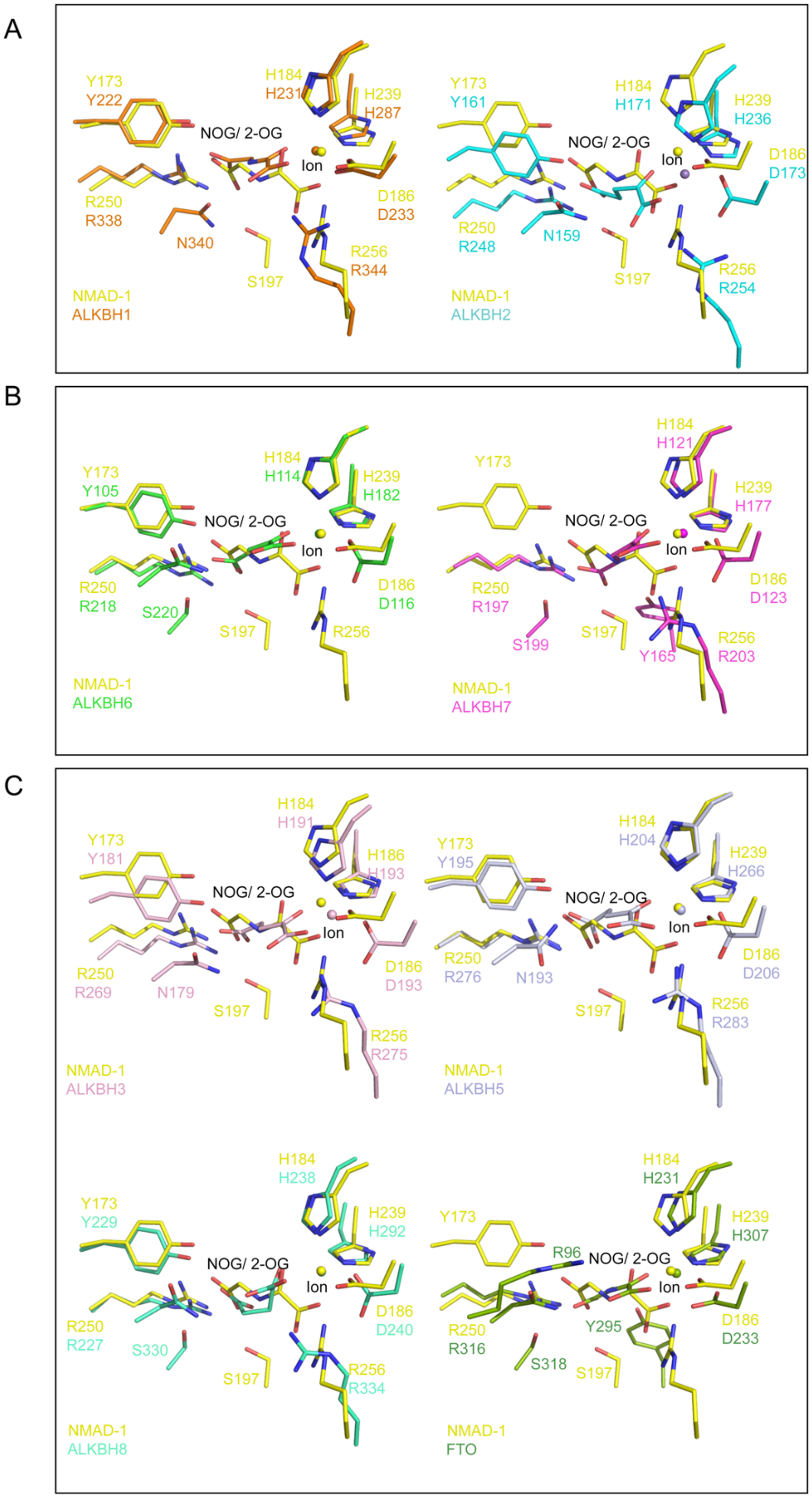

Supplementary Figure S7. Structural comparison of the highly conserved key residues involved in the metal ion- and cofactor-binding at the active site between NMAD-1 and human AlkB family

members. Structural comparison of the active sites between NMAD-1 and human AlkB family members including **(A)** double-stranded DNA demethylases (ALKBH1 and ALKBH2), **(B)** potential protein demethylases (ALKBH6 and ALKBH7), and **(C)** single-stranded DNA or RNA demethylases (ALKBH3, ALKBH5, ALKBH8, and FTO). The key residues of NMAD-1, ALKBH1 [PDB code 6IMC (M. Zhang et al., 2020)], ALKBH2 [PDB code 3S57 (Yi et al., 2012)], ALKBH6 [PDB code 7VJV (Ma et al., 2022)], ALKBH7 [PDB code 4QKF (Wang et al., 2014)], ALKBH3 [PDB code 2IUW (Sundheim et al., 2006)], ALKBH5 [PDB code 7WKV (Kaur et al., 2022)], ALKBH8 [PDB code 3THT (Pastore et al., 2012)], and FTO [PDB code 5ZMD (X. Zhang et al., 2019)] are shown in stick models and colored as indicated. The metal ions are shown as spheres and the cofactors are shown in stick models using the same color coding.

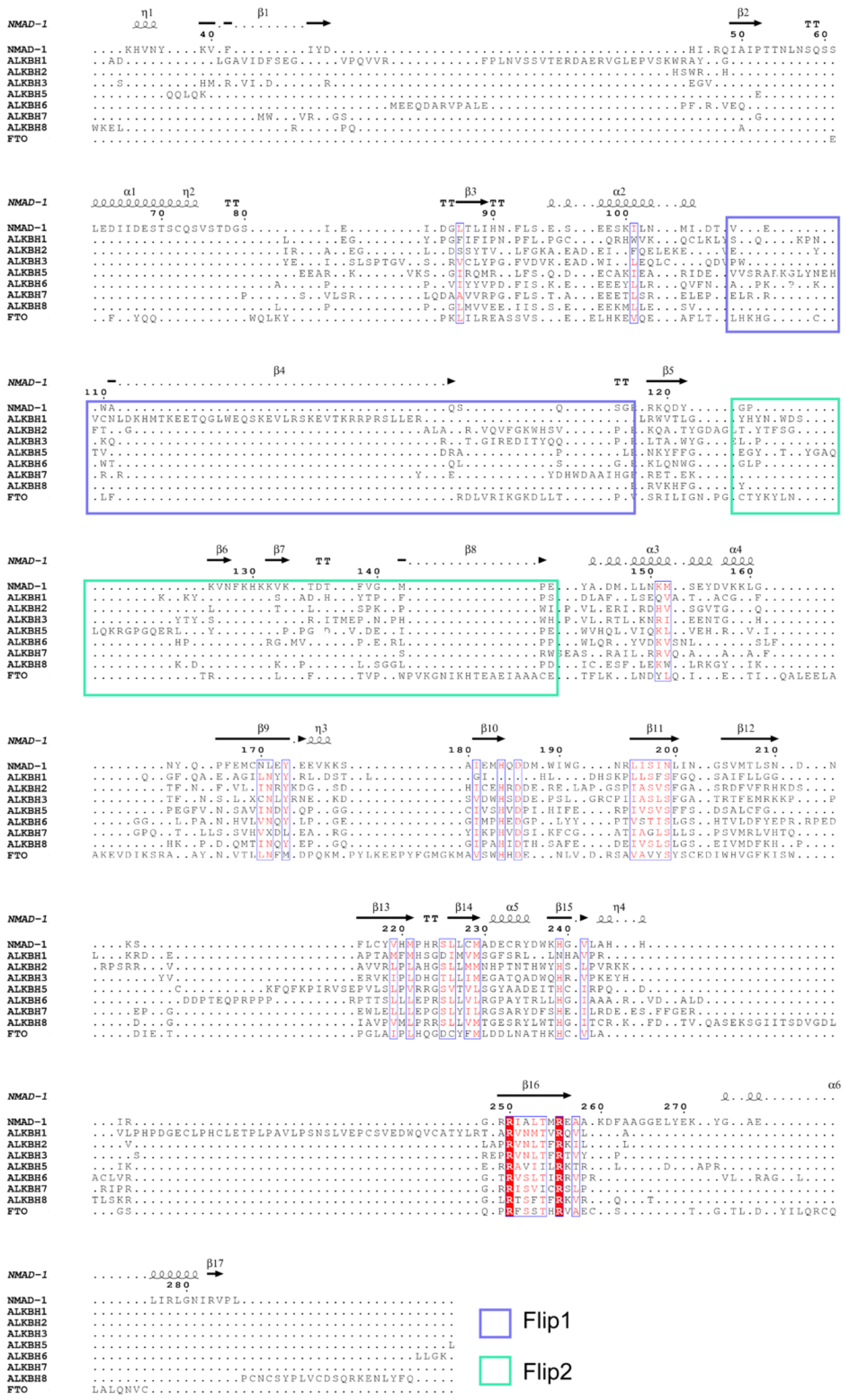

Supplementary Figure S8. **Structure-based sequence alignment of NMAD-1 and human AlkB family members.** Secondary structural elements are represented according to the structure of NMAD-$1_{32\text{-}291}$. The Flip1 and Flip2 motifs contain varied lengths of non-conserved residues and are indicated by slate and green cyan boxes, respectively.

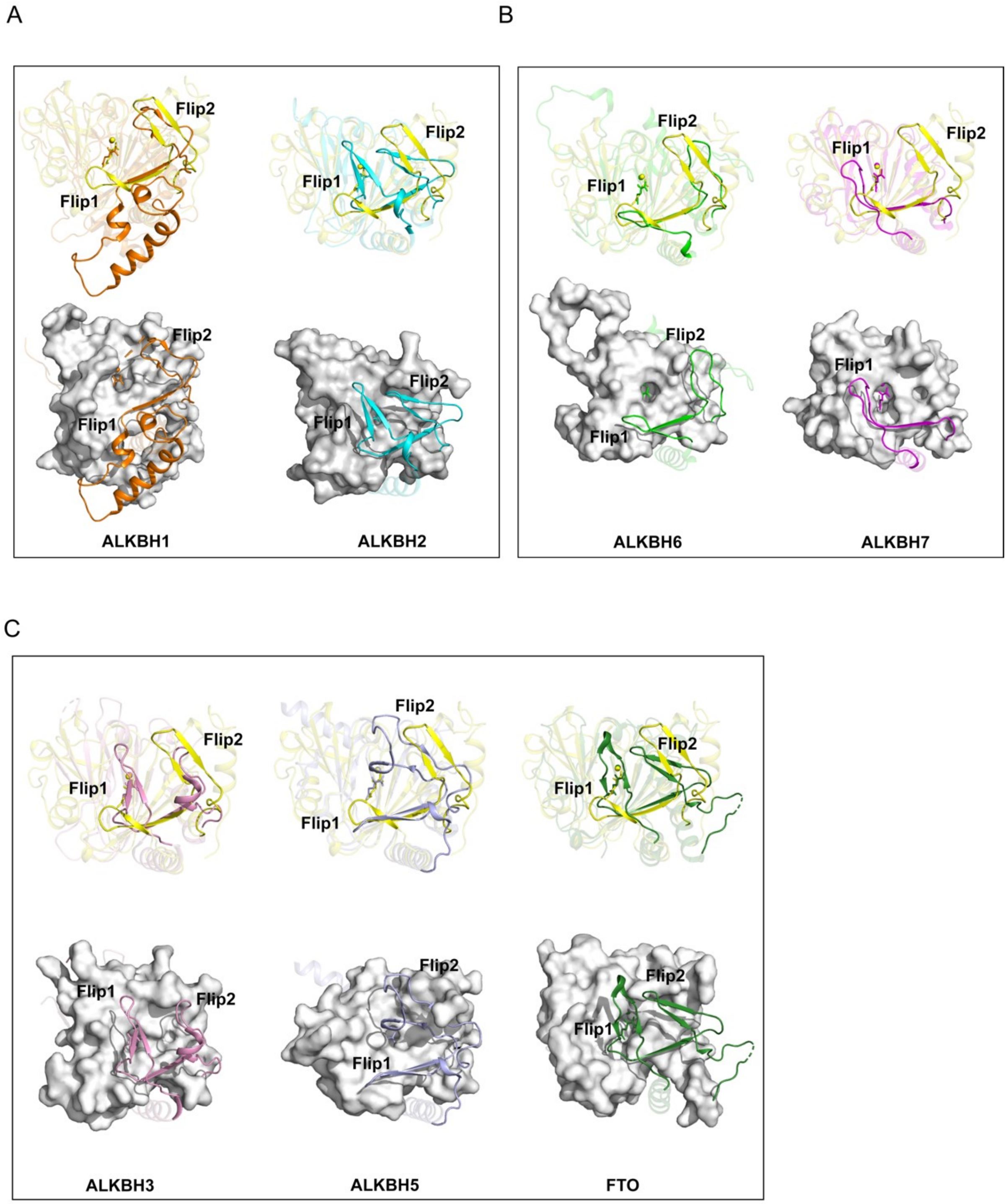

Supplementary Figure S9. **Structural comparison of the NRL domains in NMAD-1 and human AlkB family members.** Superposition of NMAD-1 on **(A)** double-stranded DNA demethylases (ALKBH1 and ALKBH2), **(B)** potential protein demethylases (ALKBH6 and ALKBH7), and **(C)** single-stranded DNA or RNA demethylases (ALKBH3, ALKBH5, and FTO), respectively. All structures and the color coding are the same as those in Supplementary Figure S7. Upper panels: Structural comparisons are shown in ribbon

presentations. Lower panels: The spatial position of the NRL domain in each AlkB protein is indicated with the DSBH domain shown as light gray surface. The NRL domain of NMAD-1 presents a distinct architecture when compared with those of human AlkB members. As the Flip1 and Flip2 motifs were partially disordered in the structure of ALKBH8, the structural comparison between NMAD-1 and ALKBH8 is not shown.

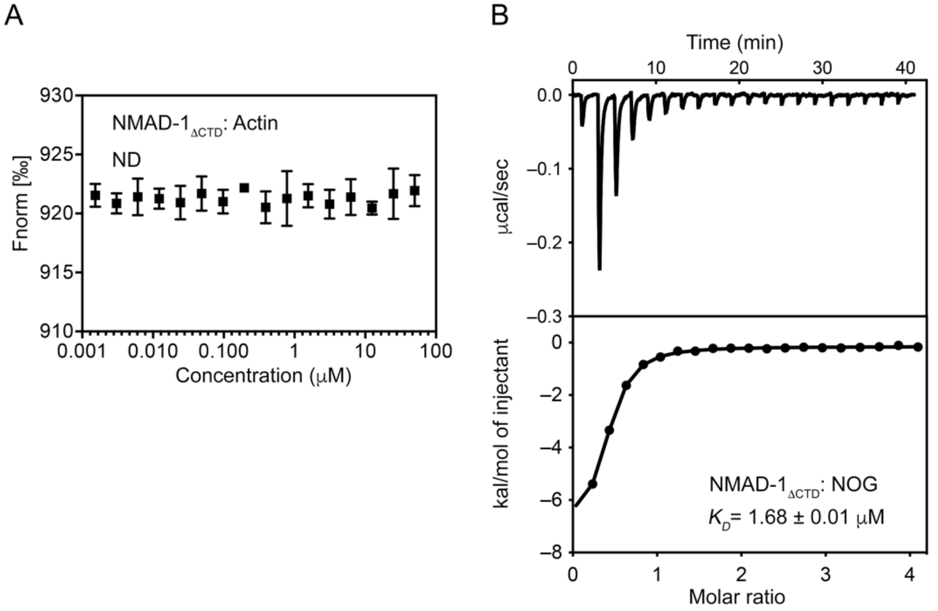

Supplementary Figure S10. **Deletion of the CTD domain interfers with the substrate binding but not the NOG binding. (A)** MST measurement of the binding affinity between the C-terminal truncated NMAD-1 (residues 1-259, ΔCTD) and the unmethylated actin. The NMAD-$1_{\Delta CTD}$ truncation exhibited no detectable binding to actin. **(B)** ITC measurement of the binding affinity of NOG to NMAD-$1_{\Delta CTD}$. The NMAD-$1_{\Delta CTD}$ truncation exhibited comparable NOG binding affinity (K_d of 1.68 ± 0.01 μM) as the wild-type NMAD-1 (K_d of 1.07 ± 0.11 μM).

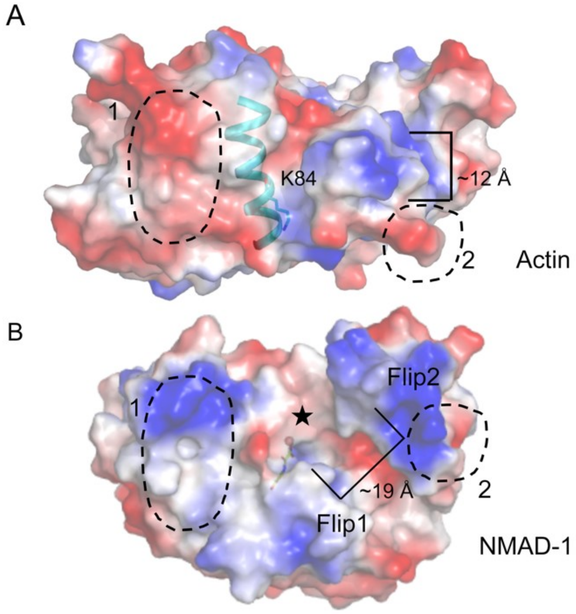

Supplementary Figure S11. **Global views of the shape and charge complementarity at the potential interacting interface between actin and NMAD-1.** Electrostatic surface representations of **(A)** human actin [PDB code 6NBW (Rebowski et al., 2020)] and **(B)** NMAD-1. Lys84 containing α-helix of human actin is shown in ribbon representation and colored in cyan, with Lys84 shown in stick model. The active site of NMAD-1 is highligted with a black star. The two poteintial interacting regions, which are indicated by dashed circles, are formed by oppositely charged surfaces from NMAD-1 and actin. The negatively charged region 1 of actin might interact with the positively charged region 1 of NMAD-1. Besides, the stretched negatively charged region 2 of actin might contact positively charged region 2 of NMAD-1. The two potentially interacting regions may facilitate the recognition of actin Lys84 by the active site of NMAD-1 and contribute to the specificity and selectivity for actin Lys84 substrate rather than other proteins. In addition, the width of the cave between Flip1 and Flip2 of NMAD-1 (~19 Å) has enough space to accommondate the bulge region of actin (~12 Å).
